# Presentación atípica de rotura de seno de Valsalva derecho aneurismático con insuficiencia aortica severa

**DOI:** 10.47487/apcyccv.v4i1.284.

**Published:** 2023-06-30

**Authors:** Miguel Ángel Serrano-Sánchez, Alfredo Sotomayor-Estrada, W Samir Cubas

**Affiliations:** 1 Servicio de Cirugía de Tórax y Cardiovascular, Hospital Nacional Hipólito Unanue, Lima, Perú. Servicio de Cirugía de Tórax y Cardiovascular Hospital Nacional Hipólito Unanue Lima Perú; 2 Departamento de Cirugía Torácica y Cardiovascular, Hospital Nacional Edgardo Rebagliati Martins, Lima, Perú. Departamento de Cirugía Torácica y Cardiovascular Hospital Nacional Edgardo Rebagliati Martins Lima Perú

**Keywords:** Senos de Valsalva, Shunt, Comunicación Interventricular, Insuficiencia Aórtica Severa, Valsalva Sinuses, Shunt, Ventricular Septal Defect, Severe Aortic Regurgitation

## Abstract

El aneurisma del seno de Valsalva (ASV) es una anormalidad poco frecuente que afecta a menos del 0,1% de la población en general. Se describe el caso de una mujer de 37 años con un cuadro clínico de 6 años de evolución caracterizado por disnea, palpitaciones y síncope. El estudio ecocardiográfico evidenció ASV derecho con una perforación interventricular subpulmonar de 8 mm que producía un *jet* de regurgitación hacia el ventrículo derecho que ocasionaba la dilatación del tracto de salida de ventrículo derecho, la arteria pulmonar e insuficiencia aórtica severa Carpentier ID. El paciente se sometió a una reparación exitosa del defecto y de la perforación interventricular, sin necesidad de reemplazo valvular. El diagnóstico ecocardiográfico oportuno y una cirugía precoz, son los principales predictores que suponen la diferencia entre una evolución excelente y una muerte segura.

## Abstract (en)

Aneurysm of the Sinus of Valsalva (ASV) is a rare abnormality and affects less than 0.1% of the general population. We describe the case of a 37-year-old woman with a clinical picture characterized by dyspnea, palpitations, and syncope of 6 years of evolution. The echocardiographic study revealed a right ASV with a sub pulmonary interventricular perforation of 8mm and producing a regurgitant jet into the right ventricle, causing dilatation of the right ventricular outflow tract, pulmonary artery, and severe aortic insufficiency Carpentier DI. The patient underwent successful repair of the defect and interventricular perforation, and valve replacement was not necessary. Timely echocardiographic diagnosis and early surgery are the main predictors that make the difference between an excellent outcome and certain death.

## Introducción

El aneurisma del seno de Valsalva (ASV) es una anormalidad poco frecuente, afecta a menos del 0,1% de la población en general, y tiene una prevalencia de 1,2-1,8% en la población china y de 0,14-0,96% en la población occidental con cardiopatía congénita ^1^^,^^2^. La edad de presentación varía ampliamente, se presenta entre los 2-74 años con una media de 39 años con predominio del sexo masculino ^2^. Se clasifican como congénitos y adquiridos, el primer grupo tiene mayor frecuencia y comprende el 3,5% de todas las cardiopatías congénitas, vinculándose además de manera muy frecuente con otros trastornos congénitos como la comunicación interventricular (en su mayoría supracrestal); la insuficiencia aórtica (4,9%); la estenosis pulmonar (9,7%); la estenosis aórtica (6,5%); la coartación aórtica (6,5%); la persistencia del conducto arterioso (3,2%), y la insuficiencia tricúspidea (3,2%) ^3^^-^^5^. Frecuentemente los ASV son derechos (75-90%), seguidos del no coronario (10 - 25%) y el resto en el seno coronario izquierdo ^2^. Suelen producirse cuando existe una alteración en la fusión entre la capa media de la pared aortica y el anillo fibroso de la válvula aórtica ^5^^-^^7^. En el grupo de los adquiridos, comparten similitudes en su localización con los de origen congénito y se han asociado en condiciones como endocarditis, sífilis, síndrome de Behcet y el síndrome de Marfan ^6^. La complicación más común que presenta este defecto es la rotura (35,6%) y ocurre de manera espontánea o después de un trauma, por ejercicio físico extremo o por endocarditis. Aunque esta última también puede ocurrir como una complicación del ASV roto (ASVR) ^8^. El ASVR ocurre en su mayoría hacia cavidades derechas del corazón, representando el 60% de los casos hacia el ventrículo derecho y en un 29% hacia aurícula derecha. Es poco frecuente que ocurra hacia el lado izquierdo o pericardio, representando aproximadamente un 10% ^5^^,^^9^. La rotura extracardíaca raramente ocurre; sin embargo, de presentarse es fatal; ocurre hacia el espacio pleural y está asociado frecuentemente con los ASVR adquiridos ^9^.

## Descripción del caso

**Figura 1:**
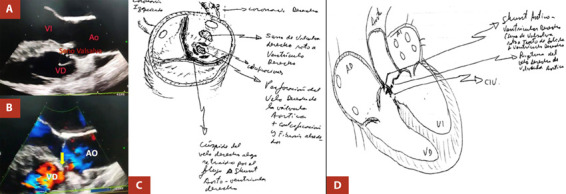
A-B. Estudio ecocardiográfico preoperatorio que muestra el ASVR de 8 mm y que presenta conexión fistulosa hacia cavidades derechas con jet de regurgitación (Flecha). C-D. Gráfico en corte transversal y coronal que muestra detalladamente los hallazgos identificados a nivel del seno coronario derecho y septum interventricular. AO=aorta, VI=ventrículo izquierdo, VD=ventrículo derecho.

Se presenta el caso de una mujer de 37 años, sin antecedentes de importancia, que ingresa a nuestra institución por presentar un cuadro clínico de 6 años de evolución caracterizado por disnea (que progresa de clase funcional I a III), palpitaciones y síncope. Al examen físico se ausculta soplo sistólico en foco aórtico (IV/VI); en el estudio ecocardiográfico se evidencia una insuficiencia aórtica severa, y aneurisma de seno del Valsalva derecho con una solución de continuidad de 8 mm. Este último produce un *jet* de regurgitación hacia el ventrículo derecho (VD), dilatando el tracto de salida del VD (TSVD) y la arteria pulmonar (AP) (Figura 1). La insuficiencia aórtica severa fue catalogada como Carpentier ID, vena contracta 0,5 cm, volumen regurgitante 70 mL; fracción regurgitarte 60%; anillo aórtico 18 mm (indexado 11,3 mm); seno de Valsalva 31 mm (indexado 19,4 mm); unión sinotubular 33 (indexado 20,7); aorta ascendente 34 mm (indexado 21,3 mm) y una función sistólica del ventrículo izquierdo conservada (FEVI) de 65%. Debido a esta condición estructural cardiaca que ocasiona una sobrecarga de cavidades derechas y ante un cuadro de falla cardiaca no compensada, se planteó la reparación quirúrgica a la brevedad.

**Figura 2:**
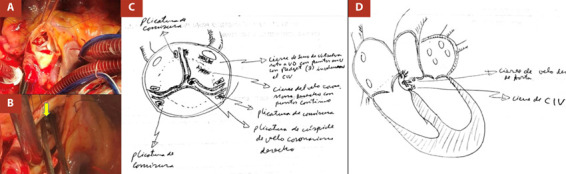
A-B. Imágenes del acto quirúrgico, confirmación de la conexión fistulosa (seno de Valsalva a Ventrículo derecho) con pinza quirúrgica (flecha). C-D. Gráfico que muestra la reparación del ASVR, cierre del CIV, plicatura comisural y de cúspide de velo coronario derecho.

Durante el acto operatorio se identificó un ASVR hacia VD; perforación de velo derecho de la válvula aortica más calcificación y fibrosis; cúspide del velo derecho retraído; *shunt* auriculoventricular derecho (seno de Valsalva a TSVD), y comunicación interventricular subpulmonar de aproximadamente 8 mm. La reparación se realizó bajo circulación extracorpórea y consistió en el cierre del ASVR a VD con puntos en «u» utilizando *pledget* más cierre de la perforación del velo coronariano derecho con puntos continuos de polipropileno 4/0; plicatura de comisuras y cúspide de velo coronariano derecho, y cierre de comunicación interventricular sin parche (Figura 2). La intervención quirúrgica se desarrolló sin ninguna complicación perioperatoria y la paciente ingresó a la Unidad de Cuidados Intensivos (UCI) para su recuperación posquirúrgica. El posoperatorio tuvo una evolución favorable, inicialmente con apoyo de antihipertensivos endovenosos e inotrópicos a dosis bajas. La ecocardiografía control reportó una FEVI de 51% asociado a una insuficiencia aortica leve y sin *shunts* residuales. Luego de 72 h en UCI, la paciente fue extubada, y pasados los 7 días de estancia hospitalaria, el nuevo control ecocardiográfico evidenció una FEVI de 62% por lo que la paciente fue dada de alta con indicación de control ambulatorio por consulta externa.

## Discusión

Los ASV constituyen una entidad poco frecuente, afecta predominantemente a varones y a población asiática en el 32% de los casos ^3^^,^^5^. Su presentación en nuestro medio es infrecuente, si incluimos el presente caso tenemos cinco reportes publicados sobre esta condición hasta el momento ^6^^-^^8^. Su principal etiología es congénita y se debe al adelgazamiento de la túnica media aórtica y a la fusión incompleta del tabique bulbar distal, que separa la arteria pulmonar de la aorta y se une al anillo fibroso de la válvula ^2^^,^^4^. Dado que el seno derecho y dos tercios del seno coronario se originan embriológicamente de dicho tabique, el defecto se localiza principalmente en estas dos localizaciones ^1^^,^^3^. Sin embargo, la afectación del seno de Valsalva izquierdo no es infrecuente y se debe sospechar de un origen adquirido, siendo la endocarditis infecciosa la causa más frecuente en este subgrupo ^5^.

Fisiopatológicamente, este defecto genera una debilidad de la pared, que en condiciones de alta presión propias de la aorta, produciría una dilatación de esta conllevando a una eventual rotura ^2^. La enfermedad cursa frecuentemente de forma asintomática, y cuando presenta sintomatología lo hace de dos maneras, la primera de forma compresiva debido a su tamaño creciente como para comprimir otras estructuras y generar isquemia o trombosis a nivel coronario y la segunda, generalmente de instauración aguda, debido a la rotura, generando un cuadro clínico variable dependiendo del lugar de fistulización (dolor torácico, taponamiento cardíaco, insuficiencia cardíaca, etc.) ^1^^-^^3^. El ASVR con fistulización hacia cavidades de menor presión, especialmente en atrio y ventrículo derecho, constituye probablemente la principal forma de presentación de esta condición ^3^^,^^5^. Cuando se instaura la conexión fistulosa suele aparecer clínica de insuficiencia cardíaca progresiva y de rápida evolución, siendo posible la auscultación de un soplo continuo en maquinaria, de alta intensidad y predominio mesocárdico ^4^.

Con relación al diagnóstico, la identificación definitiva se hace con los estudios ecocardiográficos, que evidencian la zona aneurismática y el flujo continuo a través de la fístula, esto puede ser complementado mediante una visión transesofágica ^1^. Sin embargo, debido a la baja frecuencia de los ASV, el diagnóstico correcto de las complicaciones que surgen de su rotura requiere un alto índice de sospecha y una exploración ecocardiográfica cuidadosa, como lo fue en nuestro caso. Esta exploración debe estar orientada hacia el diagnóstico diferencial con otras entidades más frecuentes que también pueden cursar con rotura de la pared aórtica, como son las complicaciones derivadas de la endocarditis infecciosa y la disección de aorta ^9^. Entre otras pruebas complementarias tenemos a la tomografía computarizada o la resonancia magnética, y estas se reservan para la evaluación de la aorta en su totalidad y la mejor caracterización del defecto, especialmente de cara al tratamiento definitivo; sin embargo, en este caso no fue necesario debido a que el diagnóstico fue identificado satisfactoriamente con el estudio ecocardiográfico. 

La literatura no ha llegado a un consenso sobre las indicaciones específicas del tratamiento, pero las principales guías actuales describen la opción quirúrgica para dilataciones aneurismáticas mayores a 55 mm en población general, 50 mm para pacientes con Marfan, 45 mm si existen factores de riesgo y finalmente 55 mm o más para pacientes con válvula bicúspide ^3^^,^^9^. Existen diversos reportes que describen la presentación inusual de los ASV, sus complicaciones y muchas posibilidades de reparación quirúrgica; sin embargo, entre muchas de sus conclusiones describen que la reparación de un ASVR se debe realizar de forma sistemática al momento de su diagnóstico ^1^^,^^5^. La cirugía precoz está indicada incluso en pacientes asintomáticos, ya que las fístulas aortoauriculares dejadas a su libre evolución presentan un pronóstico ominoso y terminan comprometiendo severamente la expectativa de vida del paciente a menos del 30% en 2 años ^1^^-^^4^. El pronóstico viene condicionado por la progresión hacia insuficiencia cardíaca y el riesgo de complicaciones graves como la endocarditis en el 21% de los casos ^3^^,^^5^^,^^10^. Solo en defectos asintomáticos y de muy pequeño tamaño podría considerarse una vigilancia estrecha trimestral con estudios ecocardiográficos, asumiendo que un porcentaje elevado de estos defectos (43,4%) requerirá intervención en el seguimiento en al menos dentro de los dos primeros años ^3^^,^^11^^,^^12^.

Este caso clínico representa el paradigma del ASV complicado con ruptura, una entidad compleja y muy poco frecuente, en la cual además de una elevada sospecha clínica, es importante el adecuado y detallado estudio ecocardiográfico y un tratamiento quirúrgico oportuno para permitir una buena supervivencia del paciente. En este caso en particular, es inusual por la coexistencia de tres lesiones a la vez como el seno de Valsalva derecho roto a ventrículo derecho, perforación de velo derecho de la válvula aortica y comunicación interventricular subpulmonar, convirtiendo nuestro caso en un verdadero desafío para su diagnóstico y manejo.
